# High performance magneto-fluorescent nanoparticles assembled from terbium and gadolinium 1,3-diketones

**DOI:** 10.1038/srep40486

**Published:** 2017-01-16

**Authors:** Rustem Zairov, Asiya Mustafina, Nataliya Shamsutdinova, Irek Nizameev, Beatriz Moreira, Svetlana Sudakova, Sergey Podyachev, Alfia Fattakhova, Gulnara Safina, Ingemar Lundstrom, Aidar Gubaidullin, Alberto Vomiero

**Affiliations:** 1 A. E. Arbuzov Institute of Organic and Physical Chemistry, Kazan Scientific Center of Russian Academy of Sciences, Arbuzov str., 8, 420088, Kazan, Russia; 2 Kazan (Volga region) Federal university, Kremlyovskaya str., 18, 420008, Kazan, Russia; 3 Kazan National Research Technological University, K. Marks str., 68, 420015, Kazan, Russia; 4 Department of Chemistry and Molecular Biology, University of Gothenburg, Kemigården4, 412 96 Gothenburg, Sweden; 5 Division of Biological Physics, Department of Physics, Chalmers University of Technology, Kemigården1, 412 96 Gothenburg, Sweden; 6 Division of Materials Science, Department of Engineering Sciences and Mathematics, Luleå University of Technology, SE-97187 Luleå, Sweden; 7 Department of Physics, Chemistry and Biology, Linköping University, 581 83 Linköping, Sweden

## Abstract

Polyelectrolyte-coated nanoparticles consisting of terbium and gadolinium complexes with calix[4]arene tetra-diketone ligand were first synthesized. The antenna effect of the ligand on Tb(III) green luminescence and the presence of water molecules in the coordination sphere of Gd(III) bring strong luminescent and magnetic performance to the core-shell nanoparticles. The size and the core-shell morphology of the colloids were studied using transmission electron microscopy and dynamic light scattering. The correlation between photophysical and magnetic properties of the nanoparticles and their core composition was highlighted. The core composition was optimized for the longitudinal relaxivity to be greater than that of the commercial magnetic resonance imaging (MRI) contrast agents together with high level of Tb(III)-centered luminescence. The tuning of both magnetic and luminescent output of nanoparticles is obtained via the simple variation of lanthanide chelates concentrations in the initial synthetic solution. The exposure of the pheochromocytoma 12 (PC 12) tumor cells and periphery human blood lymphocytes to nanoparticles results in negligible effect on cell viability, decreased platelet aggregation and bright coloring, indicating the nanoparticles as promising candidates for dual magneto-fluorescent bioimaging.

Noninvasive diagnostics provides *in situ* insight to the structural and functional features of the investigated systems and organs. The use of magnetic and optical techniques is emerging for these purposes. Improved sensing in tissues, which in turn can differentiate normal tissue from diseased one, can be achieved by using magnetic contrast agents (CA)^1^,^2^,^3^,^4^ and optical emissive probes^5^,^6^,^7^,^8^,^9^. The f-elements (rare earth elements) are outstanding candidates for this, since their chelates combine unique magnetic and luminescent characteristics^3^,^10^,^11^.

It is well known that trivalent lanthanide chelates are very good alternatives to organic luminescent stains or quantum dots in view of their extraordinary properties. Their emission bands span both the visible and near infrared (NIR) ranges and can be easily discriminated from the organic background by both emission wavelength and photoluminescence lifetime. They possess enhanced photostability, larger Stokes shift and longer excited state lifetime over conventional organic fluorophores^11^,^12^.

Typically, all lanthanide luminescent labels contain an organic chromophore, which serves as antenna or sensitizer to absorb the excitation light and to transfer this energy to the emission levels of lanthanide ions. This antenna acts as a shield for the lanthanide ion from the solvent quenching effects as well as a reactive group for coupling the chelate complex to biotargets^12^,^13^.

Having seven unpaired electrons at the valence shell, gadolinium in the form of chelates provides high positive contrast between healthy and tumor tissues^14^. Despite the proven clinical efficacy of Gd-DTPA, Gd-DOTA and other gadolinium-based molecular MRI agents, they suffer from dechelation and transmetallation processes taking place in the presence of chelating anions and endogenous cations, correspondingly^15^,^16^. At the same time, luminescent function of lanthanide-containing optical labels is quenched in presence of organic background in the biological sample due to antennae-ligands replacement^11^,^17^,^18^.

Nanoparticulate approach has attracted increasing interest in recent years^13^,^19^,^20^,^21^,^22^. Novel MRI CA based on gadolinium-containing nanoparticles show enhanced relaxation parameters compared to commercial specimens^13^,^23^,^24^,^25^,^26^. The slowed down rotation of magnetic ions is the main reason for better relaxivity of the nanoparticulate CA versus mononuclear gadolinium complexes. Enhanced permeation, retention, lowered toxicity of nanoparticulate CA contribute to the enhanced positive imaging, as well^27^,^28^,^29^.

Large number of attempts to combine the benefits of simultaneous magnetic and luminescent properties of lanthanides(III) were undertaken on oxide^30^,^31^,^32^,^33^, fluoride^34^,^35^,^36^,^37^ and phosphate^38^,^39^,^40^ matrices, targeting the development of multimodal imaging materials. However, the development of novel nanoparticulate platforms with possibility to conjoin efficient magnetic and optical performance of lanthanides is still a challenge.

The precipitation of lanthanide complexes from water miscible organic solvents to polyelectrolyte-containing water solutions under effective stirring is an example of easy and effective synthetic pathway to embed large amount of lanthanide functional complexes to the nanoparticle. The synthesis of europium-doped luminescent polyelectrolyte colloids was first described by Mustafina *et al*.^41^ and later applied to various terbium^42^, and gadolinium^43^ complexes.

Herein we report for the first time the synthesis and characterization of polystyrenesulfonate-coated nanoparticles consisting of terbium and gadolinium complexes with calix[4]arene tetra-diketone ligand. The new nanoparticles are rationally designed to simultaneously exhibit the optical properties of Tb and the magnetic behavior of Gd ions, towards bifunctional system with strong luminescence and high relaxivity. Colloidal, photophysical and magnetic studies were performed on nanoparticles with varying core composition, which is controlled by changing the Tb:Gd atomic ratio. We compared the relaxation parameters of the produced nanoparticles with commercial MRI agents. Particular attention was paid to the simplicity and universality of the strategy for the synthesis of bifunctional species. Tuning both magnetic and luminescent output of nanoparticles is possible via simple variation of composition of initial synthetic solution of lanthanides chelates. This synthetic strategy opens the door to new biomedical applications of lanthanide complexes.

## Results and Discussion

### Nanoparticle morphology

**Figure 1:**
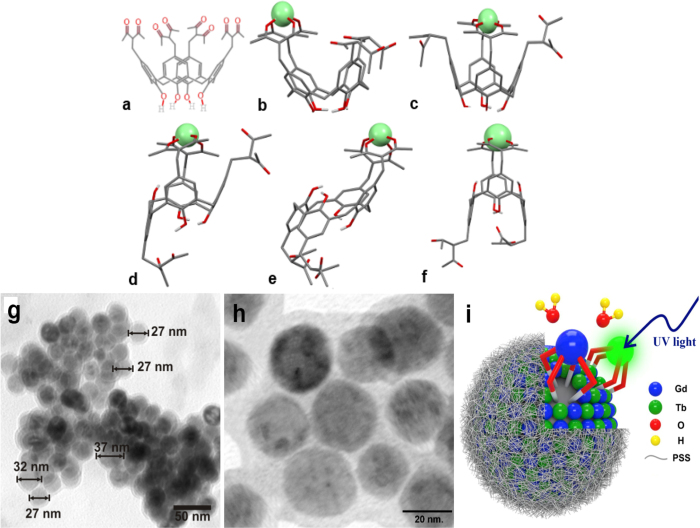
Structure of 5,11,17,23-tetrakis[(acetylaceton-3-yl)methyl)]-25,26,27,28-tetrahydroxy-calix[4]arene **1** (**a**) and possible coordination modes of Ln-**1** in the alkalized DMF solution (**b–f**). For more details see ref. 44. TEM image of dried PSS-coated Gd-**1** colloids (**g**), higher resolution TEM image of the same colloids (**h**), and schematic illustration of PSS-coated Gd-**1/**Tb-**1** nanoparticle and interactions on its core surface with water molecules and UV light (**i**).

We synthesized polyelectrolyte-coated nanoparticles consisting of terbium(III) and gadolinium(III) complexes with calix[4]arene tetra-diketone ligand **1** (Fig. 1) according to the procedure described in the experimental section. According to our previous reports, the complex formation of **1** with both Gd(III)^43^ and Tb(III)^44^ occurs in 1:1 stoichiometry in alkaline DMF solutions. Our previous report^43^ on the synthesis of PSS-stabilized Gd-**1** colloids demonstrated the ability of keeping the full amount of Gd-**1** complex within NP shell, thanks to the poor solubility of the complex, experimentally confirmed by luminescence measurements of the supernatant solutions in the synthesis of PSS-stabilized Tb-**1** colloids. This tendency provides great synthetic advantage, since the Tb-to-Gd ratio in the colloids can be easily driven by their ratio in the organic (DMF) solution. Transmission electron microscopy (TEM) and dynamic light scattering (DLS) techniques were applied to examine the morphology of the fabricated colloids. The aliquot of as-prepared colloidal solution was diluted ten times by deionized water to adjust the concentration of Ln(III) ions in the colloidal form to 0.075 mM to perform the DLS study.

The DLS measurements reveal hydrodynamic diameter being 100–170 nm in the obtained colloids (Table 1). These values are similar to those previously reported for other polyelectrolyte-coated lanthanide chelates^17^,^42^. Their size distribution is narrow even without any prefiltration or fractionizing, which is clearly seen from the polydispersity (PDI) indices. Moreover, the sizes are stable during the period of one week at least.

The molar ratio of Ln (Tb or Gd) in the core of Ln-**1** based colloids was calculated via equation (1):


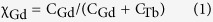


where C_Gd_ and C_Tb_ are the concentrations of the lanthanide chelates.

DLS and electrokinetic potential measurements confirm hard core-soft shell morphology of the colloids (Table 1), where the high negative charge of PSS-based exterior layer is the reason for their high colloidal stability. The DLS data reveal the size of hydrated PSS-coated colloids, while their functionality results from the hard cores inside the polyelectrolyte coating^17^,^43^,^45^. The previously published reports^17^,^43^ highlight hard-soft morphology of the colloids. The TEM image of the dried colloids presented in Fig. 1g confirms the hard-soft nature of the colloids, where soft polyelectrolyte shell is evident from grey color spheres, while denser lanthanide-based cores can be clearly defined from the darker shade of grey color. The nanoparticle diameters are in the range 27–37 nm and the polyelectrolyte shell thickness is 2–3 nm. Sizes revealed from the TEM images of the dried aqueous colloids (Fig. 1g) are lower than those estimated by DLS measurements, because of their aggregation and swelling of polyelectrolyte coating in aqueous solutions. Moreover, the hard templates within the soft PSS coating are not uniform, consisting of smaller (2–10 nm sized) cores, as previously reported for PSS-stabilized Gd-**1** colloids^43^. The TEM images of PSS-stabilized Tb-**1** colloids confirm similar morphology (Fig. 1h). Figure 1i schematically illustrates the core-shell morphology of PSS-coated Gd-**1/**Tb-**1** nanoparticle.

**Figure 2:**
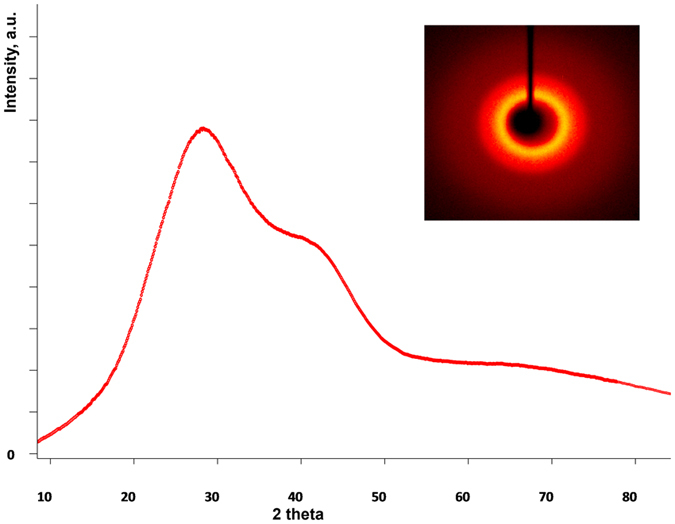
X-ray diffraction pattern of the PSS-stabilized Tb-**1** colloids. Two-dimensional X-ray diffraction picture (inset).

The size distribution of PSS-stabilized Gd-**1/**Tb-**1** colloids at various Gd content from the TEM data (Fig. S1 in SI) revealed no detectable effect of the Gd content on their size. The uniformity of both Gd- and Tb-based colloids is fully consistent with the isomorphism of Tb-**1** and Gd-**1** complexes. It is worth noting that the previously reported amorphous nature of the hard templates of PSS-stabilized Gd-**1** colloids results from metastability of the hard cores, probably due to reprecipitation of the complex from organic to aqueous solutions^43^. Similar XRD measurements (for detailed description of the procedure see SI) were performed here for PSS-stabilized Tb-**1** colloids. Both diffraction pattern and two-dimensional diffraction picture (Fig. 2) confirm the amorphous nature of the hard cores of the core-shell colloids. Moreover, XRD measurements of the ligand **1** (5,11,17,23-tetrakis[(acetylaceton-3-yl)methyl)]-25,26,27,28-tetrahydroxy-calix[4]arene) also indicate its amorphous nature (Fig. S2 in SI).

### Luminescence properties

It is known that the intraconfigurational 4f–4 f transitions are formally forbidden, and thus possess very low molar absorptivities, limiting direct excitation of lanthanide-centered emission. Complexation with a sensitizing chromophore (referred as the antenna) allows overcoming this restriction, providing indirect excitation of Ln(III) emission levels via energy transfer from singlet and triplet levels of organic ligand. 1,3-Diketones have been reported as a very promising antenna-ligands for the Ln(III)-centered emission^46^,^47^,^48^. The luminescent properties of Tb-**1** have recently been discussed^44^.

The Ln-**1** aqueous colloids with various χ_Tb_ exhibit emission under excitation at 320 nm with the spectrum patterns ^5^D_4_ → ^7^F_6_ (494 nm), ^5^D_4_ → ^7^F_5_ (545 nm), ^5^D_4_ → ^7^F_4_ (587 nm) peculiar for Tb(III)-centered luminescence (Fig. S3 in SI). The intensity of the main emission at 546 nm coming from the ^5^D_4_–^7^F_5_ transition is applied for the quantitative evaluation of the change of luminescence intensity under the variation of χ_Tb._ The gradual increase of luminescence intensity with increase of number of Tb emission centers in the core of nanoparticles is expected. This trend is observed when χ_Tb_ increases from 0.2 to 0.6, while it is less pronounced when χ_Tb_ increases from 0.6 to 1.0 (Table 2). The time resolved luminescence measurements were performed in PSS-stabilized Tb-**1**/Gd-**1** colloids at various χ_Tb_. The decay curves of Tb(III)-centered luminescence (Figs S4–S8 in SI) are well fitted by a biexponential decay. Monoexponential decay results in poorer fitting parameters. The obtained excited state lifetime values shown in Table 2 point to the presence of Tb(III)-containing species with longer (τ_1_ = 0.271–0.296 ms) and shorter (τ_2_ = 0.066–0.072 ms) values. The origin of the double τ values most probably arises from chelated and dechelated forms of Tb(III) species within PSS-stabilized Tb-**1**/Gd-**1** nanoparticles. Taking into account the size of PSS-stabilized Tb-**1**/Gd-**1** nanoparticles (Fig. 1) a dechelation of Tb(III) complexes at the interface is the most probable reason for the origin of the dechelated Tb(III) forms. The higher τ_1_ values tend to remain unchanged with the χ_Tb_ increase from 0.2 to 0.6, while gradually decrease with the increase in χ_Tb_ from 0.6 to 1.0, while the smaller τ_2_ values remain almost constant (Table 2). It is also worth noting that τ_1_ and luminescence intensity present opposite trends as a function of χ_Tb_. The decreased intensity as a function of increasing χ_Tb_ can be explained by the concentration-induced quenching of Tb(III)-centered luminescence^49^,^50^. The concentration-induced quenching occurs when the distance between emission centers is small enough for efficient cross relaxation (^5^D_3_:^7^F_6_) → (^5^D_4_:^7^F_0_) between two neighboring Tb(III) ions^51^.

The above-mentioned amorphous nature of PSS-stabilized Ln-**1** colloids (Fig. 2) confirms the dense packing of lanthanide centers within hard cores of the colloids. The obtained results reveal the optimal composition (χ_Tb_ = 0.4) of PSS-stabilized Tb-**1**/Gd-**1** nanoparticles for the best combination of steady state intensity and excited state lifetime values.

### Magnetic properties

**Figure 3:**
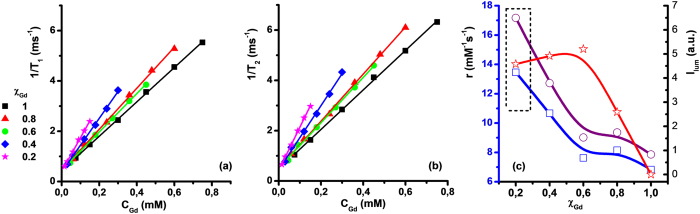
1/T_1_ (**a**) and 1/T_2_ (**b**) of PSS-stabilized Ln-**1** nanoparticles with various χ_Gd_: 1.0 (1), 0.8 (2), 0.6 (3), 0.4 (4), 0.2 (5) versus Gd(III) concentration. Straight lines are linear fitting of the experimental data. Relaxivity values r_1_ (1-blue) and r_2_ (2-purple), luminescence intensities (3-red) of PSS-stabilized Ln-1 colloids versus χ_Gd_ (**с**). Dashed rectangle reflects the optimal χ_Gd_ in the colloids core exhibiting the best magnetic and luminescent parameters.

Longitudinal and transverse relaxation times T_1_ and T_2_ of water molecule protons in the presence of studied paramagnetic Gd(III)-containing colloids were registered at 20 MHz magnetic field frequency (Tables S1–S6 in SI). The 1/T_1_ and 1/T_2_ plots versus Gd(III) concentration of PSS-stabilized Tb-**1**/Gd-**1** aqueous colloids at various χ_Gd_ are presented in Fig. 3a,b and Table 2. The corresponding longitudinal and transverse relaxivities (r_1_ and r_2_) of PSS-stabilized Ln-**1** nanoparticles were calculated as a tangent of plots’ incline angle at 1/T_1(2)_ vs C_Gd_ coordinates and presented in Fig. 3c as a function of χ_Gd_.

It is worth noting that r_1_ and r_2_ values keep almost constant, when χ_Gd_ decreases from 1.0 to 0.6. The further decrease in χ_Gd_ from 0.6 to 0.2 results in the increase of r_1_ and r_2_ (Fig. 3c). The explanation of this tendency should be preceded by discussing on main mechanisms contributing to relaxation of water protons at the colloid/water interface. Albeit no theoretical framework is known for accurate interpretation of relaxivities in Gd(III)-based colloids, several mechanisms are proposed as predominantly contributing to enhancing water protons relaxivity in Gd(III)-based aqueous colloids. Slow rotation resulting from inclusion of mononuclear Gd(III) complexes into nanoparticles should be claimed as the main reason for high relaxivity in Gd(III)-based aqueous colloids. This factor is guided by size of the hard cores inside the polyelectrolyte coating. Taking into account that exchange between inner-sphere and bulk water molecules is another key factor affecting relaxivity of Gd(III)-based colloids, size-dependent ratio of Gd(III)-centers localized close to the surface to those localized inside the hard cores is also worth noting. The size of the hard cores inside the PSS-coating varies from 2 to 10 nm for PSS-stabilized Gd-**1** colloids^43^. According to TEM results, the size of the colloids is not greatly affected by the change in χ_Gd_, although no exact assignment of the smaller versus larger nanoparticles within the polyelectrolyte coating to Tb-**1** or Gd-**1** complex, as well as their mixture, can be done. The increase in r_1_ and r_2_ for χ_Gd_ from 0.6 to 0.2 points to predominant localization of Gd(III) centers close to the colloid/water interface at low χ_Gd_ values. The reasons for this behavior are not clear, and further investigations are ongoing.

The luminescence intensities (I) of PSS-stabilized Ln-**1** colloids were plotted in Fig. 3c together with the r_1_ and r_2_ values versus χ_Gd_ in order to show the correlation between the property and the composition of the colloids. Dashed rectangle shows the optimal χ_Gd_ in the colloids core exhibiting the best magnetic and luminescent parameters.

Taking into account that both molecular and nanoparticulate (PSS-stabilized colloids) forms of Tb-**1** suffer from different radiationless decay mechanisms, it is interesting to evaluate the ratio of luminescence intensity measured in aqueous PSS-stabilized colloids of Tb-**1** (I_NP_) to that in DMF solution of Tb-**1** (I_MOL_) at the same complex concentration (0.75 mM). The measured spectra (Fig. S9 in SI) point to the stronger luminescence intensity of the colloids versus the complex in solution. The ratio I_NP_/I_MOL_ is equal to 1.7 ± 0.2, which argues in favor of application of PSS-stabilized Tb-**1** colloids versus Tb-**1** complexes as contrast agents in bioimaging.

### Effect of the colloids on cell viability and platelets aggregation

**Figure 4:**
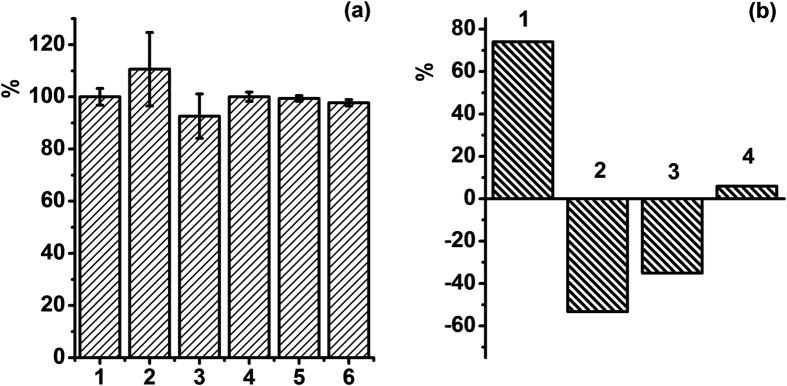
(**a**) Cell viability of periphery human blood lymphocytes without (1) and in the presence of 32 μg/mL of PSS-coated Gd-**1** (2), and 66 μg/mL of Tb-**1** (3) nanoparticles; PC 12 tumor cells (4) in the presence of 50 μg/mL of PSS-coated Tb-**1** colloids (5) and 500 μg/mL of PSS-coated Tb-**1** colloids (6). (**b**) Platelet aggregation in the presence of 10 μM adrenaline (1), 86 μg/mL PSS-coated Gd-**1** (3), and 193 μg/mL PSS-coated Tb-**1** (4) nanoparticles and without any additives (2) as a per cent of absorptivity changes at 540 nm after 5 min.

The toxic effect of PSS-stabilized Tb-**1** and Gd-**1** colloids on human blood lymphocytes and pheochromocytoma (PC12) cells were determined by means of MTT assay (based on tetrazolium dye MTT 3-(4,5-dimethylthiazol-2-yl)-2,5-diphenyltetrazolium bromide) and trypan blue viability test, respectively. The obtained results indicate no toxic effect of the colloids on human blood lymphocytes at 32 μg/mL of PSS-coated Gd-**1** colloids and 66 μg/mL of PSS-coated Tb-**1** colloids (Fig. 4a). Both 50 μg/mL and 500 μg/mL of Tb-**1** colloids show no negative effect on PC12 cell viability. The number of viable cells was calculated as 99.4% and 97.7%, respectively.

Effect of the colloids on platelet aggregation is of great importance for their biomedical applicability due to the risk of formation of blood clots. The effect of the colloids on platelet aggregation was determined in the 200 μl mixture of 50 μl of platelets mass in Hanks balanced salt solution (HBSS), and 150 μl of PBS (0.1 M, pH 7.2) in the presence of PSS-stabilized Ln-**1** colloids. The values in Fig. 4b indicate the decrease in the rates of platelet aggregation after the admixture with the colloids. The obtained results indicate low thrombogenic potential of PSS-stabilized Tb-**1** colloids, which along with their low cytotoxicity effect point to potential applicability of the colloids in bioimaging.

### Interaction of colloids with cells using fluorescent microscopy

A cellular uptake behavior of luminescent nanoparticles is affected by several factors, including size, exterior charge and aggregation of nanoparticles in physiological media^52^,^53^. Taking into account high negative electrokinetic potential values of PSS-stabilized colloids (Table 1), very poor, if any, cellular uptake behavior of the nanoparticles is anticipated due to weak interaction of negatively charged nanoparticles with cellular membrane. Nevertheless, interactions of the nanoparticles with inorganic and biological background in physiological media can affect the localization of nanoparticles on cell membrane. Thus, DLS measurements were performed for the aqueous colloids in PBS buffer before and after the protein admixture, since protein corona is well documented factor, affecting both aggregation and cellular uptake behavior of nanoparticles^54^,^55^. The size increase from ∼100 to ∼500 nm (Table 1 and S7 in SI) is observed from DLS measurements of PSS-stabilized Tb-**1** colloids in water and in PBS buffer (0.1 M). This tendency indicates that the exterior charge neutralization resulted from the binding with counterions. Aqueous buffer solutions of bovine serum albumin (BSA), which was applied as a model protein, are characterized by 10–11 nm aggregates (Table S7 in SI). The admixture of BSA (1 g·L^−1^) to PSS-stabilized colloids in the buffer solutions results in the size decrease to ∼400 nm, although this trend can be explained by the contribution of the smaller aggregates of BSA.

**Figure 5:**
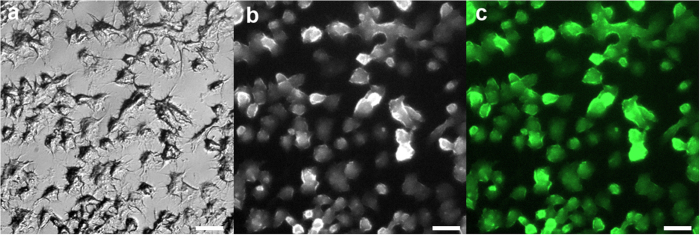
Images of PC 12 tumor cells: optical (**a**); confocal in the presence of PSS-coated Tb-**1** nanoparticles (**b**); colored rework of b using GFP emission filter (510–550 nm) (**c**), λ_ex_ = 360–370 nm. Scale bar indicates 20 um.

Figure 5 illustrates bright field and confocal fluorescent images of rat PC12 cells after their exposure for 2 hours at 37 °C to PSS-stabilized Tb-**1** colloids. The images indicate predominant localization of the emissive nanoparticles at the cellular membrane, although some cellular uptake cannot be excluded. It is worth noting that cellular uptake behavior can be enhanced by the recharging of the exterior layer of PSS-stabilized Tb-**1** colloids, which will be done in the nearest future, although the represented results highlight a potential of PSS-stabilized Tb-**1** colloids as a basis for imaging applications.

## Conclusions

Core-shell morphology nanoparticles with terbium and gadolinium complexes with calix[4]arene tetra-diketone ligand (**1**) as hard core and polystyrenesulfonate coating as soft shell as promising basis for magneto-luminescent imaging are introduced for the first time. Water dispersed polyelectrolyte-stabilized Ln-**1** nanoparticles exhibit high colloidal stability. Their size is about 30 nm in the dried state, increasing to about 100 nm in aqueous solutions due to hydration effect. Luminescent and magnetic relaxation properties of the colloids are greatly dependent on Gd-**1**:Tb-**1** ratio. The latter can be easily tuned for best bifunctional magneto-luminescent performance by simple mixing of Tb(III) and Gd(III) complexes in the initial DMF solution at the desired ratio. Both of Ln-**1** colloids are improved by appropriate variation of Gd-**1**:Tb-**1** ratio. The optimized composition (Gd-**1**:Tb-**1** = 0.2/0.8) of the colloids for optimal Tb(III)-centered luminescence and Gd(III)-based relaxivity results in better performance than the commercial Gd(III)-contrast agents. The low cytotoxicity and thrombogenic potential of PSS-stabilized Tb-**1** colloids open the door for their application in biomarking. Confocal microscopy imaging reveals predominant localization of the emissive nanoparticles at the cellular membrane, which is in agreement with the negative exterior charge of PSS-stabilized Tb-**1** colloids.

## Experimental section

### Reagents and materials

Gadolinium nitrate Gd(NO_3_)_3_·6H_2_O (99.9%) (Alfa Aesar), terbium nitrate hydrate (Tb(NO_3_)_3_∙5H_2_O) (Alfa Aesar) triethylamine (TEA) (Acros Organics), poly(sodium 4-styrenesulfonate) (PSS) (MW_average_ = 70000) (Acros Organics), sodium chloride (Sigma-Aldrich), were used as commercially received without further purification. N,N-Dimethylformamide (DMF) (Acros Organics) was twice distilled over P_2_O_5_.

Synthesis of 5,11,17,23-tetrakis[(acetylaceton-3-yl)methyl)]-25,26,27,28-tetrahydroxy-calix[4]arene was reported previously^43^.

### Synthesis of colloids

The colloids were synthesized via precipitation of water non-soluble lanthanide(III) complexes from DMF solution to polyelectrolyte containing aqueous solution. Four equivalents of triethylammonia (TEA) additives (C = 18 mM) to DMF solution of 4.5 mMcalix[4]arene 1 were followed by lanthanide(III) (C = 4.5 mM) addition to promote the formation of 1:1 complex between lanthanide(III) cations and anions of 1. The aliquote of 1 ml DMF dissolved lanthanide(III) complex was added to the 5 ml of polystyrenesulfonate sulfonate aqueous solution (1 g·L^−1^) in the presence of NaCl (C = 0.5 M)at the effective stirring (2200 rpm) dropwisely using the syringe pump. The solution becomes turbid since the dispersion of polyelectrolyte stabilized nanoprecipitate forms. The obtained colloids were washed from the DMF and excess amounts of PSS and NaCl via triple centrifugation (11000 rpm, 10 min.)/decantation and redispergation (deionized water addition and ultrasonication for 30 min.) procedures.

Six syntheses were performed from DMF solutions with different Gd/Tb molar ratios (χ_Gd_ = 0, 0.2, 0.4, 0.6, 0.8 and 1) within the core of polyelectrolyte coated colloids. Two equimolar solutions of Gd-**1** and Tb-**1** in DMF were prepared (C = 4.5 mM) for this purpose. The Gd/Tb core composition was varied via volume of aliquots of Gd-**1** and Tb-**1** in initial DMF solution. The colloids were ultrasonicated for 30 min before measurements. All measurements have been performed in triplicates.

### Methods of characterization of colloids

DLS measurements were performed using Malvern Mastersize 2000 particle analyzer operating with a He–Ne laser (633 nm) and emitting vertically polarized light as a light source. The transmission electron microscopy (TEM) images have been obtained with Hitachi HT7700, Japan. Samples have been sonicated in water for 30 min and then dispersed on 200 mesh copper grids with continuous formvar support films. The images have been acquired at an accelerating voltage of 100 kV.

The steady-state luminescence and time-resolved spectra have been recorded on a spectrofluorometer FL3–221-NIR (Jobin Yvon). Excitation of samples has been performed at 320 nm, and emission detected at 545 nm with 1 nm slit width for both excitation and emission.

Bruker “Minispec mq20” NMR analyzer was employed to measure T_1_ and T_2_ of water molecule protons in studied solutions at 20 MHz magnetic field. Inversion-recovery and Carr-Purcell-Meiboom-Gill (CPMG) pulse sequences^56^ were used for longitudinal T_1_ and transverse T_2_ relaxation times accordingly with 20 points data collected for fitting^57^.

Powder X-ray diffraction (PXRD) measurements were performed on a Bruker D8 Advance diffractometer equipped with Vario attachment and Vantec linear PSD, using Cu radiation (40 kV, 40 mA) monochromated by the curved Johansson monochromator (λ Cu K_α1_ 1.5406 Å). Room-temperature data were collected in the reflection mode with a flat-plate sample. The samples were loaded on a standard zero diffraction silicon plate, which was kept spinning (15 rpm) throughout the data collection. Patterns were recorded in the 2*Θ* range between 3° and 60°, in 0.008° steps, with a step time of 0.1–4.0 s. Several diffraction patterns in various experimental modes were collected and summed for the sample. Processing of the obtained data performed using EVA^58^ software packages. Additional experiments were carried out on a single-crystal X-ray diffractometer Bruker Smart Apex II CCD using Mo radiation (Mo*K*_*α*_, graphite monochromator, λ 0.71073 Å) at the 23 °С with the liquid samples in a standard glass 1 mm capillary. ССD-detector of the diffractometer was kept on the fixed values of 2θ angles (0° and 30°), recording time was varied from 30 to 400 seconds. Standard powder diffractograms were obtained by integration of several two-dimension scattering patterns with the use of software packages APEX2^59^.

### Cell viability test

Cell viability of human blood lymphocytes towards nanoparticles was estimated using MTT proliferative test (Promega, USA). Yellow MTT (3-(4,5-dimethylthiazol-2-yl)-2,5-diphenil-tetrazolium bromide) turns into purple formazan under the effect of mitochondrial NADN reductase of living cells. After the lysis of the cells formazan dissolves in the DMSO. The activity of the mitochondrial reductase (and cell viability respectively) has been defined as the function of the formazan absorbance (500–600 nm).

Human blood lymphocytes (7500 cells per well) were cultivated in 100 μl of DMEM in the presence of nanoparticles under the standard conditions in a 96-well cultural plate during 3.5 hours. Cells were washed using PSB (phosphate-buffered saline (PBS; 10 mM/L; pH 7.2) and the mixture of 80 μl of DMEM and 20 μl of nanoparticles was added. Then the 20 μl aliquot of 5 mg/ml MTT was added to each well and incubated in CO_2_-incubator under the 37 °C for 3.5 hours. The medium was removed then and 150 μl of DMSO were added. The absorbance of the resulted solutions was measured at 590 nm after 10 minutes using plate spectrophotometer Stat Fax 2100 (Awareness Technology, USA).

The effect of NPs on viability of PC12 cells was assessed by the trypan blue exclusion test. All NPs samples were sonicated for 30 min prior the experiment. Solutions of NPs were prepared by diluting the stock solution of NPs in cell growth media and adjusting to pH 7.4. Cells were seeded in flasks and, after confluence was reached, they were exposed for 2 h with 0 (control), 50 μg/mL and 500 μg/mL of Tb-**1** colloids. Conditions of exposure were 5% CO_2_, 37 °C, 100% humidity. Thereafter, cells were trypsinized, by standard trypsinization, and resuspended in equal volumes of culture medium and trypan. Viable (unstained) and nonviable (blue-stained) cells were counted using a haemocytometer, and cell viability was calculated as (N viable cells/N total cells) × 100%.

### Platelets aggregation

The citrated human blood was centrifuged at 120·g for 15 min at room temperature to obtain platelets mass containing 25·10^7^ of platelets per ml. Platelets concentration was determined with automatic hematology analyzer Abacus (Diatron, Austria) according to the manufacturer method.

The platelets aggregation was detected in a 200 μl mixture of 50 μl of platelets mass in HBSS (125·10^5^ of platelets), 50 μl of 0.188 mM/L water dispersion of nanoparticles and 100 μl of PBS (0.1 M, pH 7.2). Platelet aggregation was recorded after 5 min.

Positive control 200 μl mixture contained 50 μl of platelets mass in HBSS, adrenaline (10 μM, Sigma) and 100 μl of PBS (0.1 M, pH 7.2).

The spontaneous platelets aggregation was examined in a 200 μl mixture of 50 μl of platelets mass in HBSS, and 150 μl of PBS (0.1 M, pH 7.2). The absorbance of the resulted solutions at 540 nm was measured using spectrophotometer Shimadzu (Japan) at 37 °C. The extent of aggregation was calculated quantitatively as a percentage of the maximal change in optical density.

The authors confirm that all methods were carried out in accordance with relevant guidelines and regulations. A Kazan Federal university committee approved all experimental protocols. Informed consent was obtained from all volunteers. There are no animal experiments included in the project.

## Additional Information

**How to cite this article**: Zairov, R. *et al*. High performance magneto-fluorescent nanoparticles assembled from terbium and gadolinium 1,3-diketones. *Sci. Rep.* **7**, 40486; doi: 10.1038/srep40486 (2017).

**Publisher's note:** Springer Nature remains neutral with regard to jurisdictional claims in published maps and institutional affiliations.

## Supplementary Material

Supplementary Information

## Figures and Tables

**Table 1:** The hydrodynamic diameter, electrokinetic potential (ζ) and polydispersity indices (PDI) of PSS-covered polyelectrolyte nanoparticles as a function of Gd content (χ_Gd_ = 1, 0.8, 0.6, 0.4, 0.2, 0; χ_Tb_ = 1 − χ_Gd_).

χ_Gd_	Diameter (nm)	PDI	ζ (mV)
0	113.6 ± 1.6	0.168	−29.9 ± 0.1
0.2	106.4 ± 2.0	0.153	−32.6 ± 0.2
0.4	144.4 ± 3.4	0.354	−31.9 ± 0.2
0.6	166.4 ± 2.8	0.238	−33.1 ± 0.2
0.8	123.5 ± 2.2	0.168	−28.3 ± 0.1
1	101.8 ± 1.7	0.180	−29.5 ± 0.1

**Table 2:** Luminescence intensities (I), excited state lifetimes (τ), as well as r_1_ and r_2_ of PSS-stabilized Ln-**1** (Ln = Tb, Gd) nanoparticles at various χ_Tb_ and χ_Gd_.

χ_Tb_	χ_Gd_	I (a.u.)	τ_1_ (ms)	τ_2_ (ms)	r_1_	r_2_
1	0	5609000	0.271 ± 0.005	0.072 ± 0.002	—	—
0.8	0.2	4567000	0.277 ± 0.004	0.066 ± 0.002	13,46	17,17
0.6	0.4	4918000	0.284 ± 0.004	0.067 ± 0.002	10,67	12,72
0.4	0.6	5204000	0.296 ± 0.006	0.072 ± 0.003	7,62	9,02
0.2	0.8	2585000	0.291 ± 0.005	0.066 ± 0.002	8,15	9,36
0	1	3000	—		6,83	7,86
