# Supplementary material for: High performance magneto-fluorescent nanoparticles assembled from terbium and gadolinium 1,3-diketones
Source: Sci Rep. 2017 Jan 16;7:40486. doi: 10.1038/srep40486 (PMC5238420; doi:10.1038/srep40486)
Supplement: Supplementary Information [file srep40486-s1.doc]

Supplementary Information

**High performance magneto-fluorescent nanoparticles assembled from terbium and gadolinium 1,3-diketones**

Rustem Zairov^a,b*^, Asiya Mustafina^a,b^, Nataliya Shamsutdinova^a,b^, Irek Nizameev^a,c^, Beatriz Moreira^d^, Svetlana Sudakova^a^, Sergey Podyachev^a^, Alfia Fattakhova^b^, Gulnara Safina^d,e^, Ingemar Lundstrom^f, g^, Aidar Gubaidullin^a^, Alberto Vomiero^f*^

*^a^ A. E. Arbuzov Institute of Organic and Physical Chemistry, Kazan Scientific Center of Russian Academy of Sciences, Arbuzov str., 8, 420088, Kazan, Russia.*

*^b^ Kazan (Volga region) Federal university, Kremlyovskaya str., 18, 420008, Kazan, Russia.*

*^c^ Kazan National Research Technological University, K. Marks str., 68, 420015, Kazan, Russia.*

*^d^ Department of Chemistry and Molecular Biology, University of Gothenburg, Kemigården4, 412 96 Gothenburg, Sweden.*

*^e^ Division of Biological Physics, Department of Physics, Chalmers University of Technology, Kemigården1, 412 96 Gothenburg, Sweden.*

*^f^ Division of Materials Science, Department of Engineering Sciences and Mathematics, Luleå University of Technology, SE-971 98 Luleå, Sweden.*

*^g^ Department of Physics, Chemistry and Biology, Linköping University, 581 83 Linköping, Sweden.*


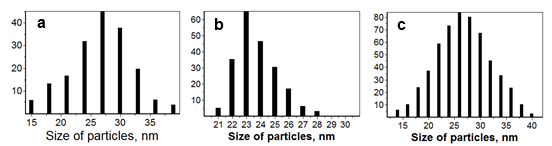


Figure S1 Size distribution histograms from TEM images (χTb = 0 (a); χTb = 0.4 (b); χTb = 1 (c)) in the core.


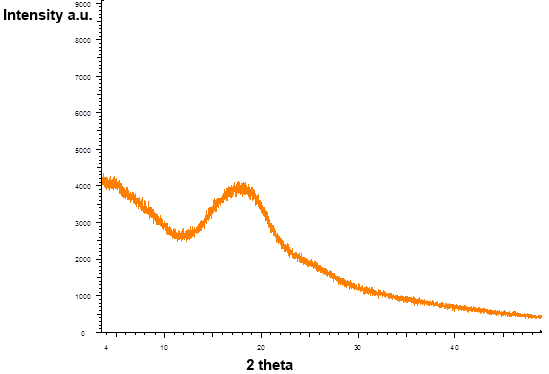


Figure S2. X-ray diffraction pattern of the ligand **1.**

Figure S3. Luminescence intensity values of PSS-coated colloids with different terbium/gadolinium ratio (χ_Tb_ = 0 (1); χ_Tb_ = 0.2 (2); χ_Tb_ = 0.4 (3); χ_Tb_ = 0.6 (4); χ_Tb_ = 0.8 (5); χ_Tb_ = 1 (6)) in the core.

Figure S4 Decay curve of PSS-stabilized Ln-**1** nanoparticles with various χ_Tb_= 1.0 (blue) and its exponential fitting (red).

Figure S5 Decay curve of PSS-stabilized Ln-**1** nanoparticles with various χ_Tb_= 0.8 (blue) and its exponential fitting (red).

Figure S6 Decay curve of PSS-stabilized Ln-**1** nanoparticles with various χ_Tb_= 0.6 (blue) and its exponential fitting (red).

Figure S7 Decay curve of PSS-stabilized Ln-**1** nanoparticles with various χ_Tb_= 0.4 (blue) and its exponential fitting (red).

Figure S8 Decay curve of PSS-stabilized Ln-**1** nanoparticles with various χ_Tb_= 0.2 (blue) and its exponential fitting (red).

Figure S9. Luminescence intensity of 0,75 mM aqueous solution of PSS-[Tb-**1]** colloids (1) and 0,75 mM DMF solution of Tb-**1** (1).

Table S1. Longitudinal relaxation time (T_1_) and rate (1/T_1_), and transverse relaxation time (T_2_) and rate (1/T_2_) of one layered PSS-covered polyelectrolyte nanoparticles on the basis of Gd-**1**.

| С(Gd-**1**), mM | С(Gd), mM | Т_1_, ms | Т_2_, ms | 1/T_1_,ms^-1^ | 1/T_2_,ms^-1^ |
| --- | --- | --- | --- | --- | --- |
| 0.75 | 0.75 | 180.7 | 158.3 | 5.53 | 6.32 |
| 0.6 | 0.6 | 220.0 | 192.9 | 4.55 | 5.18 |
| 0.45 | 0.45 | 281.1 | 243.0 | 3.56 | 4.12 |
| 0.3 | 0.3 | 410.4 | 352.7 | 2.44 | 2.84 |
| 0.15 | 0.15 | 682.1 | 612.5 | 1.47 | 1.63 |
| 0.075 | 0.075 | 1061 | 962.5 | 0.94 | 1.04 |

Table S2. Longitudinal relaxation time (T_1_) and rate (1/T_1_), and transverse relaxation time (T_2_) and rate (1/T_2_) of one layered PSS-covered polyelectrolyte nanoparticles on the basis of Ln-**1** (Ln=Tb, Gd; Gd-**1**/Tb-**1**=0.8/0.2).

| С(Ln-**1**), mM | С(Gd), mM | Т_1_, ms | Т_2_, ms | 1/T_1_,ms^-1^ | 1/T_2_,ms^-1^ |
| --- | --- | --- | --- | --- | --- |
| 0.75 | 0.6 | 189.4 | 163.9 | 5.28 | 6.10 |
| 0.6 | 0.48 | 226.2 | 198.8 | 4.42 | 5.03 |
| 0.45 | 0.36 | 291.5 | 255.9 | 3.43 | 3.91 |
| 0.3 | 0.24 | 426.3 | 375.4 | 2.35 | 2.66 |
| 0.15 | 0.12 | 676.8 | 603.8 | 1.48 | 1.66 |
| 0.075 | 0.06 | 1121 | 930.3 | 0.89 | 1.07 |

Table S3. Longitudinal relaxation time (T_1_) and rate (1/T_1_), and transverse relaxation time (T_2_) and rate (1/T_2_) of one layered PSS-covered polyelectrolyte nanoparticles on the basis of Ln-**1** (Ln=Tb, Gd; Gd-**1**/Tb-**1**=0.6/0.4).

| С(Ln-**1**), mM | С(Gd), mM | Т_1_, ms | Т_2_, ms | 1/T_1_,ms^-1^ | 1/T_2_,ms^-1^ |
| --- | --- | --- | --- | --- | --- |
| 0.75 | 0.45 | 259.6 | 218.0 | 3.85 | 4.59 |
| 0.6 | 0.36 | 312.5 | 268.8 | 3.2 | 3.72 |
| 0.45 | 0.27 | 396.6 | 341.2 | 2.52 | 2.93 |
| 0.3 | 0.18 | 538.8 | 467.4 | 1.86 | 2.14 |
| 0.15 | 0.09 | 870 | 696.6 | 1.15 | 1.44 |
| 0.075 | 0.045 | 1345 | 1215.2 | 0.74 | 0.82 |

Table S4. Longitudinal relaxation time (T_1_) and rate (1/T_1_), and transverse relaxation time (T_2_) and rate (1/T_2_) of one layered PSS-covered polyelectrolyte nanoparticles on the basis of Ln-**1** (Ln=Tb, Gd; Gd-**1**/Tb-**1**=0.4/0.6).

| С(Ln-**1**), mM | С(Gd), mM | Т_1_, ms | Т_2_, ms | 1/T_1_,ms^-1^ | 1/T_2_,ms^-1^ |
| --- | --- | --- | --- | --- | --- |
| 0.75 | 0.3 | 275.4 | 230.8 | 3.63 | 4.33 |
| 0.6 | 0.24 | 344.8 | 288.7 | 2.90 | 3.46 |
| 0.45 | 0.18 | 444.5 | 374.7 | 2.25 | 2.67 |
| 0.3 | 0.12 | 591.4 | 507.7 | 1.69 | 1.97 |
| 0.15 | 0.06 | 948.0 | 744.4 | 1.05 | 1.34 |
| 0.075 | 0.03 | 1446 | 1304.1 | 0.69 | 0.77 |

Table S5. Longitudinal relaxation time (T_1_) and rate (1/T_1_), and transverse relaxation time (T_2_) and rate (1/T_2_) of one layered PSS-covered polyelectrolyte nanoparticles on the basis of Ln-**1** (Ln=Tb, Gd; Gd-**1**/Tb-**1**=0.2/0.8).

| С(Ln-**1**), mM | С(Gd), mM | Т_1_, ms | Т_2_, ms | 1/T_1_,ms^-1^ | 1/T_2_,ms^-1^ |
| --- | --- | --- | --- | --- | --- |
| 0.75 | 0.15 | 418.3 | 336.9 | 2.39 | 2.97 |
| 0.6 | 0.12 | 494.5 | 398.1 | 2.02 | 2.51 |
| 0.45 | 0.09 | 604.0 | 500.4 | 1.66 | 1.99 |
| 0.3 | 0.06 | 846.0 | 698.5 | 1.18 | 1.43 |
| 0.15 | 0.03 | 1269 | 1038.5 | 0.79 | 0.96 |
| 0.075 | 0.015 | 1726 | 1510.8 | 0.60 | 0.66 |

Table S6. Longitudinal relaxation time (T_1_) and rate (1/T_1_), and transverse relaxation time (T_2_) and rate (1/T_2_) of one layered PSS-covered polyelectrolyte nanoparticles on the basis of Tb-**1**.

| С(Ln-**1**), mM | С(Gd), mM | Т_1_, ms | Т_2_, ms | 1/T_1_,ms^-1^ | 1/T_2_,ms^-1^ |
| --- | --- | --- | --- | --- | --- |
| 0.75 | 0 | 1881 | 886.0 | 0.53 | 1.13 |
| 0.6 | 0 | 1951 | 995.7 | 0.51 | 1.00 |
| 0.45 | 0 | 2136 | 1181.5 | 0.47 | 0.85 |
| 0.3 | 0 | 2184 | 1359.7 | 0.46 | 0.74 |
| 0.15 | 0 | 2500 | 1800.8 | 0.40 | 0.56 |
| 0.075 | 0 | 2514 | 2178.6 | 0.39 | 0.46 |

Table S7. DLS data of PSS-coated Tb-**1** colloids in PBS (0.1 M) and PBS-based BSA (1 g·L^-1^) solutions.

|  | Diameter  (nm) | PDI |
| --- | --- | --- |
| PSS-Tb-**1** in PBS | 532.3±11.7 | 0.936 |
| PSS-Tb-**1** in PBS-BSA | 448.6±5.7 | 1.000 |
| PBS-BSA | 10.97±3.4 | 0.344 |
